# Diversity of floral regulatory genes of *japonica* rice cultivated at northern latitudes

**DOI:** 10.1186/1471-2164-15-101

**Published:** 2014-02-05

**Authors:** Laura Naranjo, Manuel Talón, Concha Domingo

**Affiliations:** 1 Instituto Valenciano de Investigaciones Agrarias, Carretera Moncada-Naquera Km 4,5, Moncada 46113, Spain

**Keywords:** Flowering, Short day, Rice, Polymorphism, Natural variation

## Abstract

**Background:**

Rice is considered a short day plant. Originally from tropical regions rice has been progressively adapted to temperate climates and long day conditions in part by modulating its sensitivity to day length. *Heading date 3a* (*Hd3a*) and *RICE FLOWERING LOCUS T 1* (*RFT1*) that code for florigens, are known as major regulatory genes of floral transition in rice. Both *Hd3a* and *RFT1* are regulated by *Early heading date 1* (*Ehd1)* and *Days to heading on chromosome 2* (*DTH2*) while *Heading date 1* (*Hd1*) also governs *Hd3a* expression. To investigate the mechanism of rice adaptation to temperate climates we have analyzed the natural variation of these five genes in a collection of *japonica* rice representing the genetic diversity of long day cultivated rice.

**Results:**

We have investigated polymorphisms of *Hd3a*, *RFT1*, *Ehd1, Hd1* and *DTH2* in a collection of 57 *japonica* varieties. *Hd3a* and *RFT1* were highly conserved, displaying one major allele. Expression analysis suggested that *RFT1* rather than *Hd3a* could be the pivotal gene controlling flowering under long day conditions. While few alleles were found in the *Ehd1* promoter and *DTH2* coding region, a high degree of variation in *Hd1*, including non-functional alleles, was observed. Correlation analysis between gene expression levels and flowering periods suggested the occurrence of other factors, additionally to *Ehd1,* affecting *RFT1* regulation in long day adapted cultivars.

**Conclusions:**

During domestication, rice expansion was accompanied by changes in the regulatory mechanism of flowering. The existence of non-functional *Hd1* alleles and the lack of correlation of their presence with flowering times in plants grown under long day conditions, indicate a minor role of this branch in this process and the existence of an alternative regulatory pathway in northern latitudes. Expression analysis data and a high degree of conservation of *RFT1* suggested that this gene could be the main factor regulating flowering among *japonica* cultivars adapted to northern areas. In the absence of inhibition exerted by *Hd1* through repression of *Hd3a* expression, the role of *Ehd1* as a regulator of *RFT1* and *Hd3a* appears to be reinforced. Data also indicated the occurrence of additional regulatory factors controlling flowering.

## Background

Modern rice (*Oryza sativa* L.) originated in southern China and, accompanying human migration, was expanded to a wide range of geographical regions [1] diverging into *indica*, *japonica* and *aus* types. These genetic groups are characterized by specific climate adaptation, according to the agroecological conditions where they were cultivated. The *indica* genotypes are grown in tropical climates whereas *japonica* genotypes, as they suffered a wider expansion, can be found either in tropical or temperate climates. The adaptation process to new climate conditions involved changes in the genome that conferred advantages against adversity and, hence, selected and transmitted through generations. Reaching northern limits of its natural cultivation area, *japonica* rice was progressively adapting to short growing seasons. In these new areas, flowering under long days (LD) allowed cultivation in summer thus avoiding cold winter temperatures. Genes involved in flowering regulation should show allelic differentiation across environmental gradients and the frequency in the allele pool present in different geographical regions may reflect the mechanism of adaptation of plants to new conditions of day length.

The regulation of flowering is a fine tuned mechanism that changed as part of the adaptation process to new climate conditions. Two independent photoperiod pathways, involving the floral regulators *Heading date 1* (*Hd1*) and *Early heading date 1* (*Ehd1*), have been implicated in flowering date. These pathways converge through modulation of the expression of *Heading date 3a* (*Hd3a*) or *RICE FLOWERING LOCUS T1* (*RFT1*), the genes that encode florigen, a mobile signal that moves from the leaves to the apical meristem to switch on the flowering process [2,3]. *Hd1* is predominantly regulated by the circadian clock through *OsGIGANTEA* (*OsGI*)*,* a key gene that positively controls *Hd1* expression [4]. Hd1 is a zinc finger type transcriptional activator with a conserved CCT-domain that acts as a crucial nuclear localization signal for the correct protein function [5]. It has been proposed that the presence of light-active phytochromes could modify Hd1 activity to inhibit *Hd3a* transcription [6]. Under SD conditions, Hd1 activates *Hd3a* transcription thereby promoting flowering, whereas under non-inductive LD conditions Hd1 acts as a repressor [6,7]. This claim is supported by the fact that loss-of-function *Hd1* mutants display early and late heading dates when grown under LD and SD conditions, respectively [8]. Hd1 protein can also be modified in the presence of Heading date 6 protein (Hd6) suppressing *Hd3a* expression, but only when Hd1 is functional [9]. Hd6 shows casein kinase activity, but there is evidence that Hd6 does not phosphorylate directly Hd1 suggesting the action of an additional factor. *Ehd1* is unique to the flowering pathway in rice and codes for a *B*-type response regulator that, in an *Hd1*-independent manner, induces flowering under both SD and LD conditions [10]. Expression analysis using transgenic plants over-expressing *Ehd1* indicate that *Ehd1* activates transcription of *Hd3a* in rice, as well as other additional rice orthologs of Arabidopsis *FT* genes [10]. *Ehd1* is thought to be a signal integrator of floral transition as multiple regulatory pathways convey through *Ehd1* to regulate *Hd3a* or *RFT1* expression. In part, *OsGI*, the circadian clock mediator*,* regulates *Ehd1* expression under SD conditions via *OsMADS51*[11]. *OsMAD50*, another MADS box gene, is a positive regulator of *Ehd1* expression, and it works either parallel with or downstream of *OsGI*[12,13]. *Ghd8*/*DTH8*, was identified as a major QTL with pleiotropic effects on grain yield, heading date and plant height. This QTL appears to delay flowering under LD conditions, and promote it in the opposite conditions. Moreover, *Ghd8/DTH8* also represses the expression of *Ehd1* and *Hd3a* under LD conditions, but it is not clear if the QTL acts directly on *Hd3a* or down-regulates *Ehd1*[14,15]. It is also known that *Oryza sativa LEC1 and FUSCA-LIKE 1* (*OsLFL1*) and *Ghd7* delays flowering under LD conditions, most likely repressing *Ehd1*[16,17]. In a different pathway, *Ehd2/OsId1* up-regulates *Ehd1* expression under both SD and LD conditions [18,19]. Early heading date 4 (Ehd4) protein is localized in the nucleus and has acid-binding and transcriptional activation properties. Furthermore, Ehd4 promotes flowering increasing *Ehd1* expression [20]. On the other hand, phytochromes inhibit flowering by affecting both *Hd1* and *Ehd1* regulation. *PHOTOPERIOD SENSITIVITY5* (*SE5*), for example, that encodes a heme oxygenase involved in the biosynthesis of the chromophore part of phytochrome, represses *Ehd1* expression and therefore acts as a floral repressor [21].

It has been shown that the expression pattern of *Hd1* is clearly distinct from that of *Ehd1. Hd1*, which functions as a circadian clock mediator, shows similar patterns of expression under both SD and LD conditions whereas *Ehd1* is mainly induced under SD conditions [10]. These observations imply that, in rice, *Hd1* and *Ehd1* are antagonistic under LD, but are synergistic under SD. *Hd1*, depending upon phytochrome abundance, promotes flowering under SD and inhibits flowering under LD, whereas *Ehd1* promotes flowering under both conditions [10,22]. It has been suggested that the equilibrium between the influence of both pathways that converge on *Hd1* and *Ehd1* results in *Hd3a* modulation and, consequently, in the regulation of floral transition under SD conditions [22].

*Hd3a* is a member of the *FT*-like family and shares 91% of identity with *RFT1*, the closest homologue. Although there is evidence that *FT-L1/FTL* is possibly involved in flowering [6,23], *Hd3a* and *RFT1* are considered to be the major contributors of the FT-like members to floral promotion since suppression of both *Hd3a* and *RFT1* expression results in no flowering [23]. Although it has been demonstrated that *RFT1* can certainly activate flowering in the absence of *Hd3a* expression under SD conditions, a secondary role however has been given to *RFT1* in floral promotion under SD conditions [23]. On the other side, *RFT1* seems to be a major floral activator under LD conditions as evidenced by RNAi studies where expression of either *Hd3a*, *RFT1* or both was suppressed [3]. In addition, this is supported by the fact that *SDG724*, a rice histone methyltransferase gene, that is required for flowering under LD conditions modulates positively *EHd1* and *RFT1* expression, but not *Hd3a*[24]. Despite all these major factors involved in the flowering regulation, some other factors affecting florigen regulation should be considered. Recently, *Days to heading on chromosome 2 (DTH2)*, a minor-effect quantitative trait locus that promotes heading under LD conditions has been cloned. *DTH2* encodes a CONSTANT-like protein that promotes flowering by inducing *Hd3a* and *RFT1* independently of *Hd1* and *Ehd1*[25].

Diversification of flowering time was one of the major reasons for rice expansion. Selection of favorable alleles during the domestication and breeding process lead to the generation of new varieties adapted to different climate conditions. In this regard, the identification and characterization of allele diversity in rice populations, offers information of relevance on the mechanism of adaptation of these plants. Studies on variation of flowering regulatory genes have previously been performed on the three pivotal genetic factors contributing to flowering time in rice under SD: the promoter type of *Hd3a*, the functionality of *Hd1* and the expression level of *Ehd1*[26,27]. These studies indicated that changes in flowering time are probably not dependent on the intrinsic features of the florigens, but rather on their regulation. Hd3a protein function has been shown to be highly conserved among rice cultivars since no functional changes were found in Hd3a in either cultivated or ancestral rice. Additionally, *Hd3a* expression level showed a strong correlation with flowering time under SD conditions [27,28], suggesting that differences in the *Hd3a* promoter type, rather than protein variation, directly affects the variation in *Hd3a* expression levels. Similarly to *Hd3a*, *Ehd1* also encodes a highly conserved protein in cultivated rice. Although full evidence is still lacking, there are indications that variations in genes functioning upstream of *Ehd1* in the regulatory pathway, such as *Ghd7* or *MADS50*, could potentially provide variations in *Ehd1* expression [27,29]. In contrast, a high degree of polymorphism has been found in *Hd1*, that possesses several non-functional alleles. Loss-of function alleles of *Hd1* are common in cultivated rice and it has been suggested that they cause flowering time diversity [28]. Analysis of variation of *Hd1* in modern and ancient cultivars indicated that multiple introgression events in certain alleles of *Hd1*, including non-functional alleles, played a role in adaptation to specific areas at the beginning of domestication [30].

Despite all the information available, most of variation analyses of flowering regulatory genes have been performed with *indica* rice while little is known on the diversity in *japonica* varieties adapted to LD conditions. Additionally, the characterization of the allele pool could reveal information about the role of *RFT1* on induction of flowering in northern latitudes. To better understand the mechanism of flowering of *japonica* rice, cultivated in Northern latitudes adapted to LD conditions, in this study we analyzed the allele distribution and the expression of the master genes, *RFT1* and *Hd3a*, and their immediately regulators, *Hd1, Ehd1* and *DTH2*.

## Results

### Cycle length of *japonica* cultivars

**Figure 1:**
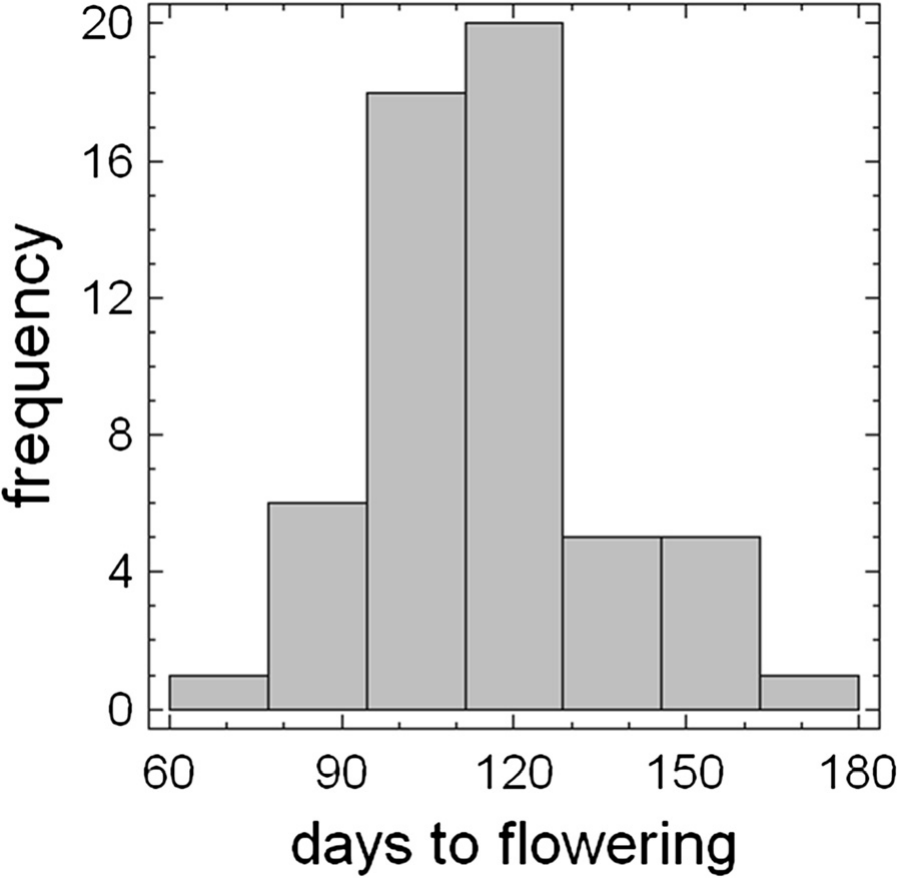
**Distribution of the mean flowering time.** Frequency distribution of the mean flowering time in *japonica* rice core collection grown under natural long day.

To analyze the genetic pool related to flowering control in *japonica* rice adapted to LD conditions, a core collection of 57 selected cultivars was used. The collection included *japonica* type cultivars from different geographical origins, mainly from Northern latitudes, and two *indica* cultivars, as control. Cultivars were representative of all genetic groups found across Northern latitudes according to recent population structure analysis [31]. Plants grown under natural LD conditions during summer were able to reach the reproductive stage and to produce panicles, except Kinmaze plants. Plants flowered ranging from 73 days to 168 days after sowing and had an average panicle emergence of 115 days after sowing (Figure 1).

### Expression analysis of *RFT1* and *Hd3a*

**Figure 2:**
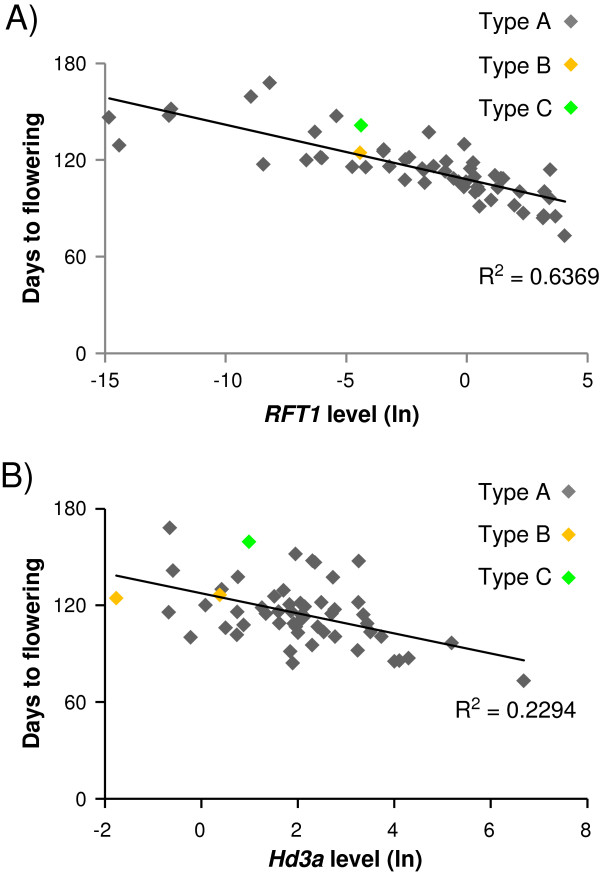
**Correlation of flowering time with RNA levels of *Hd3a *or *RFT1.*** Correlation of flowering time with RNA levels of **(A) ***RFT1* or **(B) ***Hd3a* in leaves. RNA levels were determined by real-time RT-PCR and shown as natural logarithms. The square of Pearson’s product–moment correlation coefficient is indicated (*P* < 0,05). Color dots indicate cultivars carrying different types of *RFT1* or *Hd3a* alleles.

To study the role of *RFT1* and *Hd3a* in the induction of flowering in *japonica* cultivars under LD conditions, we searched for possible relationships between their expression levels and flowering times by performing an expression analysis in 11 weeks old plants of our core collection. Expression levels of both *RFT1* and *Hd3a* were variable among cultivars. mRNA levels of *RFT1* were most closely correlated with flowering time, indicating that higher expression levels were associated with earlier flowering time (n = 56, R^2^ = 0,6369) (Figure 2A). *Hd3a* mRNA levels showed little correlation with flowering time of the different cultivars (n = 56, R^2^ = 0,2294) (Figure 2B), suggesting that *Hd3a* expression has lower effect on flowering regulation of *japonica* rice cultivated under LD conditions. Therefore, *RFT1* seems to contribute as the main factor in the regulation of transition from vegetative to reproductive phase under LD conditions among *japonica* cultivars while *Hd3a*, also expressed, participates with a minor role. These data are consistent with previous results showing that *RFT1* is a major floral activator in rice grown under LD conditions [3].

### Nucleotide polymorphism in the promoter of *RFT1* and *Hd3a*

**Figure 3:**
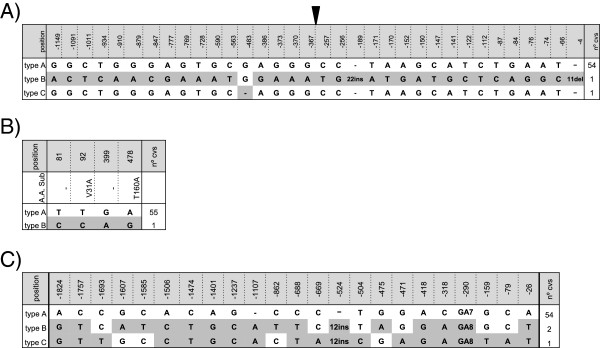
***RFT1 *and *Hd3a *are conserved among cultivated rice.** Nucleotide polymorphism in the *RFT1* promoter **(A)**, *RFT1* coding region **(B)** and *Hd3a* promoter **(C)**. The nucleotide sequences in the cultivars were compared with those of Nipponbare (indicated as Type A). Polymorphic nucleotides are indicated in grey. Numbers indicate position from ATG start codon. Amino acid substitutions caused by nucleotide polymorphism are indicated. The number of cultivars containing each type of allele is indicated in the right column. Arrow head indicates the start of the 5’-untraslated region.

We investigated the occurrence of nucleotide polymorphisms in the *RFT1* promoter region including the 5’-untraslated region (UTR) and found a high degree of conservation (Figure 3A). One major allele was shared by all cultivars in the core collection, except by two of them. One of the alleles differed only in a single nucleotide deletion. Kasalath, included in the core collection as a control, contained a third allele showing differences in the promoter region, including the UTR, consisting in single nucleotide substitutions, a deletion of 11 bp and a insertion of 22 bp. Despite variations in *RFT1* promoter in Kasalath, the expression levels of *RFT1 *followed the tendency observed in other cultivars and did not appear to affect expression (Figure 2A). As little is known about variability of the coding region of *RFT1*, we further investigated this region. We detected the presence of a unique allele among all *japonica* cultivars. Again, Kasalath showed differences that, in this case, implicated two amino acid changes in the encoded protein (Figure 3B).

Previous studies found little variability in the protein encoded by *Hd3a* and variations found in the coding region did not affect the functional domains of the protein [4,27]*.* To investigate the diversity in the promoter region of *Hd3a* in *japonica* cultivars, we analyzed in the core collection over 2-kb region of *Hd3a* including the promoter and the 5_ UTR region by EcoTilling. Nucleotide polymorphism analysis revealed that *Hd3a* was also highly conserved among *japonica* cultivars (Figure 3C). All cultivars included in this study shared the same *Hd3a* promoter sequence with the exception of three that contained two different alleles. As *RFT1*allele, Kasalath contained a different *Hd3a* allele to the rest of the core collection (Table 1). Variations identified in the *Hd3a* promoter consisted of single nucleotide substitutions, small deletions and a 12 bp insertion. No major alterations in potential regulatory sequences of the *Hd3a* promoter were identified. Within the analyzed promoter fragment, no changes affected the CCAAT box or the three binding sites recognizing the GARP DNA-binding domain of *Ehd1* that potentially can bind the *Hd3a* promoter via the ARR1 binding element [10]. The variability found in *Hd3a* promoter among the analyzed cultivars was lower than in previous studies using *indica* cultivars [27]*.*

**Table 1:** Flowering times and allele types of genes analyzed in the cultivars of the core collection

	**Variety**	**Country**		**Days to flowering**	***Hd3a *** **promoter sequence**	***RFT1 *** **promoter and coding sequence**	***Hd1 *** **coding sequence**	***Ehd1 *** **promoter sequence**	***DTH2 *** **coding sequence**
1	Agami	Egypt	Africa	168,0 ± 1,5	a	a	9	a	a
2	Moroberekan	Guinea	Africa	147,5 ± 5,2	a	a	9	b	a
3	GZ178	Egypt	Africa	116,0 ± 1,4	a	a	2	c	b2
4	Hwayoung	Korea	Asia	107,7 ± 2,3	a	a	1	a	a
5	Koshihikari	Japan	Asia	106,0 ± 6,1	a	a	1	a	a
6	Nipponbare	Japan	Asia	147,3 ± 13,7	a	a	1	a	a
7	Akihikari	Korea	Asia	113,8 ± 1,9	a	a	3	a	a
8	Sasanishiki	Japan	Asia	121,3 ± 2,9	a	a	3	a	a
9	Daegudo	Korea	Asia	91,3 ± 0,5	a	a	4	a	a
10	Dongjin	Korea	Asia	125,3 ± 14,6	a	a	4	a	a
11	Kinmaze	Japan	Asia	>190	a	a	4	a	a
12	Tainung 67	Taiwan	Asia	137,3 ± 6,2	a	a	4	a	b1
13	Zonghua 15	China	Asia	151,8 ± 4,6	a	a	4	a	a
14	Taichung 65	Taiwan	Asia	159,5 ± 0,6	c	a	5	a	d
15	Guweoldo	Korea	Asia	137,5 ± 3,7	a	a	6	a	a
16	LTH	China	Asia	101,5 ± 2,1	a	a	11	a	b1
17	Azucena	Philippines	Asia	141,5 ± 1,9	a	c	9	b	a
18	Kasalath	India	Asia	124,5 ± 4,1	b	b	8	c	b2
19	Norin 8	Japan	Asia	126,5 ± 1,0	b	a	8	c	b2
20	IR64	Philippines	Asia	129,3 ± 0,5	a	a	9	c	b2
21	Harra	Australia	Australia	115,5 ± 6,1	a	a	4	b	a
22	Guadiamar	Spain	Europe	102,8 ± 1,5	a	a	3	a	a
23	Bahia	Spain	Europe	121,8 ± 11,3	a	a	4	a	a
24	Bendret	Spain	Europe	119,0 ± 2,9	a	a	4	a	a
25	Benisants	Spain	Europe	113,0 ± 2,3	a	a	4	a	a
26	Gavina	Spain	Europe	103,3 ± 8,5	a	a	4	a	a
27	Ullal	Spain	Europe	114,8 ± 1,0	a	a	4	a	a
28	crlb1	Italy	Europe	85,0 ± 2,0	a	a	9	a	a
29	Gladio	Italy	Europe	100,5 ± 2,4	a	a	9	a	a
30	Ripallo	Spain	Europe	103,3 ± 1,9	a	a	9	a	a
31	Carnice Precoce	Italy	Europe	100,5 ± 1,9	a	a	10	a	a
32	Romolo	Italy	Europe	106,5 ± 3,0	a	a	10	a	a
33	Gigante Vercelli	Italy	Europe	87,0 ± 2,0	a	a	11	a	a
34	Giov. Marchetti	Italy	Europe	84,0 ± 0,0	a	a	11	a	a
35	Loto	Italy	Europe	85,5 ± 2,4	a	a	11	a	a
36	Bomba	Spain	Europe	118,5 ± 1,9	a	a	12	a	b1
37	Italica Livorno	Italy	Europe	106,5 ± 5,4	a	a	13	a	a
38	Pegonil	Spain	Europe	117,3 ± 1,5	a	a	13	a	a
39	Arroz da Terra	Portugal	Europe	73,0 ± 2,0	a	a	14	a	a
40	Fonsa	Spain	Europe	95,3 ± 0,5	a	a	4	b	a
41	JSendra	Spain	Europe	109,8 ± 2,2	a	a	4	b	a
42	Leda	Spain	Europe	129,8 ± 6,4	a	a	4	b	a
43	Senia	Spain	Europe	108,8 ± 8,2	a	a	4	b	a
44	Sequial	Spain	Europe	92,0 ± 3,6	a	a	4	b	a
45	Campino	Portugal	Europe	146,5 ± 1,9	a	a	9	b	a
46	Cormorán	Spain	Europe	115,8 ± 3,3	a	a	9	b	a
47	Gleva	Spain	Europe	116,3 ± 2,6	a	a	9	b	a
48	Puntal	Spain	Europe	120,3 ± 3,1	a	a	9	b	a
49	Cesare	Italy	Europe	108,5 ± 2,9	a	a	11	b	a
50	Paulovski	Russia	Europe	96,5 ± 3,0	a	a	14	b	b1
51	Calmochi-101	USA	America	100,0 ± 2,9	a	a	3	a	a
52	M201	USA	America	121,8 ± 3,8	a	a	4	a	a
53	M202 / Thainnato	USA	America	108,5 ± 3,4	a	a	4	a	a
54	L202 /Thaibonnet	USA	America	120,0 ± 2,6	a	a	9	a	a
55	Lemont	USA	America	114,0 ± 5,8	a	a	9	b	a
56	Gema	Puerto Rico	America	114,8 ± 2,4	a	a	7	b	a
57	CT-18	Colombia	America	110,5 ± 1,3	a	a	11	b	a

Flowering times and alleles types classification of *Hd3a*, *RFT1*, *Ehd1*, *Hd1* and *DTH2* in each cultivar of the core collection based on polymorphisms.

### Nucleotide polymorphism in *Ehd1*, *Hd1* and *DTH2*

**Figure 4:**
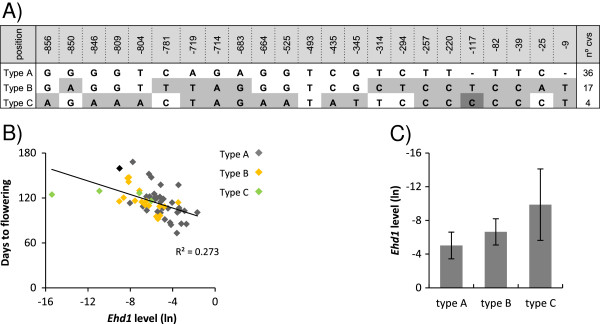
**Nucleotide polymorphisms in *Ehd1 *promoter and its relationship with flowering time. (A)** Nucleotide polymorphism in the *Ehd1* promoter. The nucleotide sequences in the cultivars were compared with those of Nipponbare (indicated as Type A). Polymorphic nucleotides are indicated in grey. Numbers indicate position from ATG start codon. The number of cultivars containing each type of allele is indicated in the right column. **(B)** Correlation of flowering time with RNA levels of *Ehd1* in leaves. RNA levels were determined by real-time RT-PCR and shown as natural logarithms. The square of Pearson’s product–moment correlation coefficient is indicated (*P* < 0,05). Color dots indicate cultivars carrying different types of *Ehd1* alleles. **(C)***Ehd1* RNA levels in cultivars with type A, B or C *Ehd1* promoters. RNA levels represented the mean and are shown as natural logarithms. Error bands represent standard deviation.

In a search for nucleotide polymorphisms that may cause differences in *Ehd1* expression, we analyzed 1 kb of *Ehd1* promoter region by EcoTilling. We identified 3 types of sequences among *japonica* cultivars (Figure 4A); although only two of them were present predominantly. Type A was shared by 36 cultivars while type B was found in 17 cultivars, and both types were widely distributed along all continents (Table 1). Type C was exclusively present in four cultivars; two of them were *indica* type and a third had *indica* ancestors suggesting that allele C is characteristic of *indica* rice. Differences between the three types of alleles consisted of single nucleotide substitutions or insertions. Expression analyses showed that *Ehd1* mRNA levels varied among cultivars and showed moderate correlation with the length of cycle (n = 56, *R*^2^ = 0,273) (Figure 4B), indicating that other factors than *Ehd1* were regulating flowering promotion under LD conditions. Additionally, despite the extent of variation, allele type A tended to show higher expression levels than allele B or C as shown in Figure 4B and C, indicating the possibility of association between promoter type and expression level.

**Figure 5:**
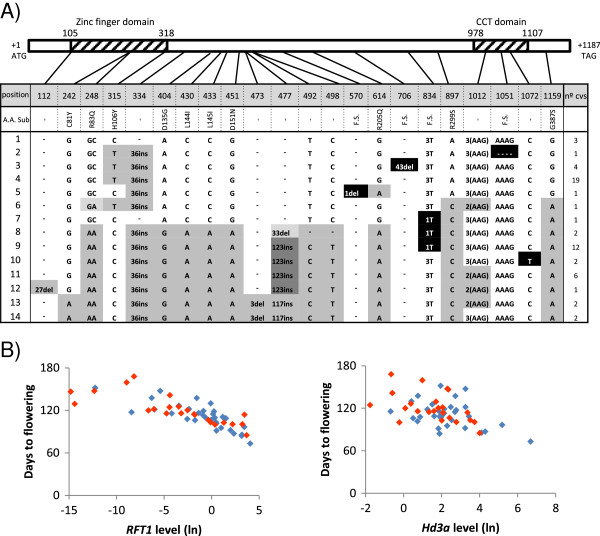
**Variations in *Hd1 *and its relationship with *Hd3a *expression levels. (A)** Nucleotide polymorphism in the *Hd1* coding region. The nucleotide sequences in the cultivars were compared with those of Nipponbare (indicated as Type 1). Polymorphic nucleotides are indicated in black or grey. Amino acid substitutions caused by nucleotide polymorphism are indicated, frame shift is indicated as F.S. Polymorphisms labeled in black caused loss of function alleles. Numbers indicate position from ATG start codon. The number of cultivars containing each type of allele is indicated in the right column. Shaded boxes represent the Hd1 zinc finger domain and CCT domain. **(B)** Correlation of flowering time with *RFT1* and *Hd3a* RNA levels of cultivars carrying functional (blue dots) or non-functional *Hd1* alleles (red dots). RNA levels were determined by real-time RT-PCR and shown as natural logarithms.

To look for changes in Hd1 that may alter the capacity of function of the protein, we analyzed polymorphisms in *Hd1* coding sequence among the core collection and we found a high degree of variation, leading to the identification of 14 different alleles (Figure 5A). Almost half of the analyzed cultivars, 23 out of 57 cultivars, contained *Hd1* variants that are likely to contain non-functional alleles of *Hd1*. Most of the loss of function was due to deletions that caused frame-shift mutations or changes in one nucleotide that created premature stop codons. Additionally, a three base pair deletion localized within the CCT-domain of the protein that acts as a nuclear localization signal was present in ten cultivars. In these varieties, the correct function of Hd1 could be affected [5]*.* Other variants affecting other regions of the protein could also cause changes in the protein activity. This is the case of a deletion of 9 amino acids at the N-terminus of the zinc finger domain in allele 12, that may cause deregulation in Hd1 binding capacity or alterations in the protein recognition capacity. C81Y substitution, in alleles 13 and 14, also affects the zinc finger domain. On the other hand, R83Q and H106Y changes have been reported previously, but no effect on the Hd1 function was detected [27]. *Hd1* alleles were distributed randomly throughout all geographical regions (Table 1), regardless of whether they carry functional or nonfunctional *Hd1* alleles.

To determine whether variations in the flowering time of *japonica* cultivars grown under LD conditions were correlated with the allelic diversity of *Hd1*, we grouped all the cultivars into those with functional and non-functional *Hd1* alleles and compared their *RFT1* and *Hd3a* mRNA levels with flowering times (Figure 5B). No correlation could be found (n = 56, P > 0,05) neither between *RFT1* or *Hd3a* mRNA levels in cultivars, carrying functional or non-functional *Hd1* alleles, and flowering time. High or low levels or mRNA were found in cultivars independently of the functionality of the displayed *Hd1* allele. These results suggested a minor influence of *Hd1* on *RFT1* and *Hd3a* regulation in flowering induction among the analyzed cultivars grown under LD conditions. This irrelevant role of *Hd1* would be in accordance with the presence of a high number of *Hd1* variants compared to *RFT1*, *Hd3a* and *Ehd1*, the other genes implicated in flowering pathway regulation. This is reinforced by the fact that there was no significant difference in flowering time between cultivars displaying functional or non-functional *Hd1* alleles, (flowering on average 110,4 ± 17,7 or 122,1 ± 20,2 days after showing, respectively).

**Figure 6:**
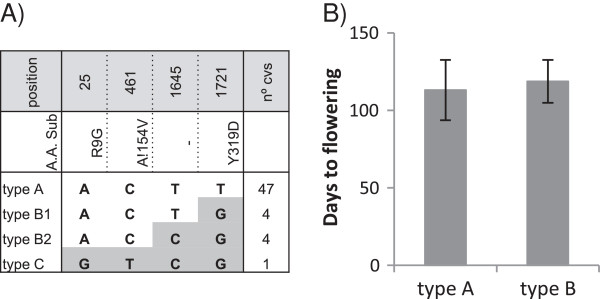
**Variations in *DTH2 *and its relationship with *flowering time. *(A)** Nucleotide polymorphism in the *DTH2* coding region. The nucleotide sequences in the cultivars were compared with those of Nipponbare (indicated as Type A). Polymorphic nucleotides are indicated in grey. Amino acid substitutions caused by nucleotide polymorphism are indicated. Numbers indicate position from ATG start codon. The number of cultivars containing each type of allele is indicated in the right column. **(B)** Days to flowering of the cultivars carrying *DTH2* allele type A or B. Data represented the mean and the error bands the standard deviation.

To investigate diversity in the *DTH2* that may cause differences in flowering time among the core collection, we examined *DTH2* coding region and we found variations in four nucleotides. Type A was present in 47 cultivars (Figure 6A). Type B1 and B2 differed in a synonymous SNP at position 1.645 and, thus, they were grouped as type B, and was found in 8 cultivars. Type C was present only in one cultivar and contained a SNP that implicated an amino acid substitution (R9G) that affected the zinc finger domain at the N-terminal region of the protein [25]. We analyzed flowering time of *japonica* cultivars grown under LD conditions carrying *DTH2* type A or type B alleles and found no significant differences (P > 0,1) in flowering time between both groups of cultivars (Figure 6B).

## Discussion

Rice is considered a short day plant, as its optimal flowering conditions are given under short day photoperiod. Rice was domesticated in two independent events in two locations in China, in a tropical region, and then it was extended to the rest of the world [1]. The spread of rice to a wide range of geographical areas encompassed adaptation to habitats with different climates that included large differences in photoperiod. The transition of rice from vegetative to reproductive stage under SD conditions is dictated by *Hd3a*, the master gene that codes for one of the mobile signal of flowering [2]. *Hd1* and *Ehd1* are the main regulators of *Hd3a* expression, acting in an independent manner. *Ehd1* is a positive regulator of flowering while Hd1 protein acts as a negative regulator under LD conditions. Thus, the balance between *Ehd1* and Hd1 function dictates flowering transition through *Hd3a* induction. The adaptation of rice to LD conditions has drawn an alternative regulatory pathway to avoid inhibition exerted by Hd1. One possibility is that an alternative gene, other than *Hd3a*, orchestrates the phase transition under LD condition. *RFT1* has been pointed as the major floral activator under LD conditions [3]. *RFT1* is a member of the FT-like family and shares 91% of identity with *Hd3a*, the closest homologue. Suppression of both *Hd3a* and *RFT1* resulted in no flowering in plants grown under SD conditions, indicating that *Hd3a* and *RFT1* are the major floral activators in rice under SD conditions [23]. According to current indications, it is believed that *Hd3a*, but not *RFT1*, is the pivotal factor promoting floral transition under SD while, *RFT1* would play a secondary role in these conditions [23]. In our core collection *RFT1* expression showed higher correlation than *Hd3a* with days to flowering in plants grown under LD conditions. These data support the hypothesis that *RFT1* is the major contributor to flowering under LD conditions in *japonica* cultivars. Additionally, the possibility that the expression of both *RFT1* and *Hd3a* affects each other has been investigated, but there is evidence that neither *Hd3a* mRNA nor Hd3a protein acts directly on the *RFT1* promoter [23]. There are common regulation factors recognizing DNA binding sites present in both promoter regions, as ARR1 or CAAT-box containing factors. In that sense, it is known that *Ehd1* regulates positively both *Hd3a* and *RFT1*[3], and it explains that *Hd3a* is expressed under LD condition in the absence of *Hd1* inhibition. It has been described that *SDG724* action specifically affects DNA methylation and differential H3K36me2/3 deposition levels at the *RFT1* locus but not at the *Hd3a* locus [24]. Chromatin modification by *SDG724* also induces expression of *Ehd1*, via *MADS50*, independently of *RFT1*[24]. *SDG274* could be a common factor of regulation of both *Hd3a* and *RFT1*, acting in an indirect manner by inducing the expression of *Ehd1* and also in a direct manner on *RFT1*.

*DTH2* modulates *Hd3a* and *RFT1* expression independently of *Ehd1* and *Hd1*. Although it represents a minor-effect quantitative trait locus it has been suggested that *DTH2* played an important role in adaptation to specific environments differing in day length [25]. Distribution of *DTH2* alleles in cultivated rice in Asia indicated that *DTH2* has been a target of artificial selection during domestication under LD conditions. We found a high degree of conservation of *DTH2* in our core collection. Most of the cultivars analyzed in this study carried the same allele previously found in *japonica* cultivars grown in Asia [25], supporting this hypothesis. We also found a second allele in our core collection that contained a polymorphism that caused an amino acid substitution that did not affect the functional domains of the protein and we found no evidence that it may cause any differences in the flowering time.

As expected, *RFT1* and *Hd3a* are well conserved among the core collection. In the *japonica* cultivars there is a major allele for each gene. It is thought that *RFT1* and *Hd3a* were generated by gene duplication followed by divergence [32]. Phylogenetic trees of *FT*-like genes showed that *RFT1* is unique to rice and that it diverged more rapidly than *Hd3a* during rice evolution [32]. Gene duplication usually generates functional redundancy and disruption by mutation of the function of one of both genes may lead to a pseudogene. Therefore, two genes with identical functions are unlikely to be stably maintained in the genome, unless both duplicates differ in some aspects of their functions [33,34]. Both *RFT1* and *Hd3a* encode the florigen signal but it appears that they have different roles since their regulation differs according to day length. In that sense, the regulation of flowering time through *RFT1* regulation may be part of a mechanism of adaptation of plants to temperate climate conditions. Functional *Hd3* gene is still present in *japonica* cultivars and it would explain the capacity to flower under SD conditions of most of the cultivars included in the core collection (unpublished observations).

*Hd1* is a repressor of flowering under LD conditions through inhibiting *Hd3a* expression. Loss-of function alleles of *Hd1* are common in cultivated rice and it has been suggested that the lack of functionality of *Hd1* has been a target during domestication to diversify flowering times [28]. We have found 14 different alleles among 57 cultivars, almost half of them are non-functional due to small deletions that caused frame shift or early stop codons. The non-functional alleles were widely distributed across all continents and they were not clearly associated with any location in particular. Similar results were found in previous studies that showed that loss-of function alleles represented more that 50% of the alleles found in *japonica* and *indica* cultivars and 95,5% in *aus* cultivars [30]. The high number of *Hd1* alleles and the presence of non-functional alleles reflect the relaxation of pressure exerted on this gene, probably due to the lower implication of *Hd1* in flowering under LD conditions and the replacement of its influence by other regulating factors. Furthermore, we have not found statistical differences between the presence of functional alleles and the time of flowering. In previous studies performed mainly with *indica* rice under SD conditions, cultivars with non-functional *Hd1* alleles showed significantly later flowering times than those with functional *Hd1* alleles [27]. However, in our core collection we did not observe any difference in flowering time among *japonica* cultivars that could be associated with the presence of a functional or non-functional *Hd1* allele. Additionally, our results indicated that *Hd3a* and *RFT1* expression levels are not affected by the type of *Hd1* allele present in each cultivar. This hypothesis appears to be reinforced if *RFT1* is considered the main regulator of floral transition. In such model, the influence of *Hd3a* and *RFT1* in flowering is specific of day length conditions and *Hd3a* becomes the major influential factor under SD conditions while *RFT1* plays this role under LD conditions. All together indicates that in the cultivars *Hd1* may not be a dominant element determining flowering transition through *Hd3a* under LD. The predominant role of *RFT1* in flowering induction in plants adapted to LD would represent an advantage by avoiding the inhibitory action of *Hd1* and, thus relaxing the evolutionary pressure on this gene. In the absence of inhibition by *Hd1*, the activation of *RFT1* and *Hd3a* would be exerted by the action of *Ehd1,* the flowering signal integrator*,* although other factors implicated in the regulation of *RFT1,* as chromatin modification by *SDG724,* may also play controlling roles.

## Conclusions

Rice was domesticated in a region located in the tropics, from where it extended to other geographical areas reaching latitudes with temperate climate where photoperiod conditions were not favorable for flowering and, thus, for reproduction. To adapt to the new conditions, rice had to overcome or avoid the inhibition action of *Hd1* under LD length. In these circumstances, two alternatives may be adopted: the emergence of non-functional *Hd*1 alleles that could inhibit flowering under LD conditions and the appearance of an alternative regulatory pathway governed by *RFT1*. The existence of non-functional *Hd1* alleles and the lack of correlation of *Hd3a* expression levels with the flowering time of the cultivars adapted to LD conditions support the hypothesis that *Hd3a* plays a minor role in the regulatory pathway in northern latitudes. Additionally, our data suggested that *RFT1* could be the main factor in the regulation of flowering among *japonica* cultivars adapted to LD conditions. In the absence of inhibition exerted by *Hd1* through repression of *Hd3a*, the influence of *Ehd1* in the regulation of *RFT1* and *Hd3a* appeared reinforced. The data also indicated the occurrence of other regulatory factors controlling both master genes.

## Methods

### Plant material and growth conditions

The rice core collection was obtained from different germplasm collections: the International Rice Research Institute (IRRI, Philippines), U.S. National Plant Germplasm System (NPGS, USA), Rice Genome Resource Center (RGRC, Japan) and Instituto Valenciano de Investigaciones Agrarias (IVIA, Spain). Seeds from different cultivars were germinated and grown in pots in greenhouses (39° 28’ N) under controlled temperature (25°C) and RH (50% RH) and natural daylight conditions during summer and were thereafter exposed to more than 13 hour daylight during the whole cycle. Four to 6 independent plants were used to score flowering time. The emergence of the first panicle from the node was recorded and referred as flowering time.

### EcoTilling analysis

To detect allelic polymorphisms present in each cultivar, seeds were germinated and leaf tissue from 15 days seedlings was frozen and DNA extracted, using DNeasy plant mini kit (Qiagen). Specific primers were designed to amplify form 1 to 1,5 kb fragments of *Hd3a*, *RFT1*, *Ehd1* or *Hd1* (Additional file 1: Table S1)*.* DNA from each cultivar was amplified and the individual PCR products were mixed with IR64 or Nipponbare PCR amplification products in equal amounts and, after denaturing and re-naturing to allow the formation of heteroduplex DNA molecules, they were digested with endonuclease CelI, isolated from celery. Fragments were separated and analyzed by electrophoresis using agarose gels. Cultivars were grouped according to the observed electrophoretic patterns and DNA fragments from five or six cultivars representing each group were sequenced to determine polymorphisms.

### RNA isolation and quantitative real-time PCR assays

Total RNA was isolated with the RNeasy plant mini kit (Qiagen), followed by DNase digestion. The RNA concentration was determined by a fluorometric assay with the kit Quant-iT^TM^ RiboGreen® RNA Assay (Molecular Probes Inc, OR). One-step Real-time PCRs were performed as previously described y on a LightCycler® 2.0 (Roche Applied Science, GE Healthcare) using the LightCycler® Fast Start DNA MasterPlus Sybr Green I kit (Roche Applied Science, GE Healthcare). One hundred nanograms of total RNA were used for each analysis. Primers used for the analysis are indicated in Supplemental Table 1. The RT-PCR procedure consisted in incubation at 48°C for 30 min, followed of 45 cycles at 95°C for 2 s, 58°C for 8 s and 72°C for 13 s. The values presented are the mean of two biological replicates, each with two technical replicates. The error bars indicate the standard deviation from the mean.

### Statistical analysis

Statistical analyses were performed using Statgraphics Plus or Microsoft Office Excel 2007. Correlation between flowering time and gene expression level was examined by Pearson’s correlation coefficient test. Comparison between flowering times and *Hd3a* expression levels of plants with functional and non-functional Hd1 alleles were determined by the 2-tailed student’s test. Differences with P < 0.01 were considered to be significant.

## Abbreviations

bp: Base pair; LD: Long day; ln: Natural logarithm; mRNA: Messenger RNA; RT-PCR: Real-time polymerase chain reaction; SD: Short day.

## Competing interests

The authors declare that they have no competing interests.

## Authors’ contributions

CD conceived the project, designed research, and wrote the manuscript. LN performed EcoTilling analysis, RNA isolation and expression analysis, interpretation of data and participated in manuscript preparation. MT designed research, participated in manuscript preparation and revision. All authors read and approved the final manuscript.

## Supplementary Material

Additional file 1: Table S1 Sequences of primers used in EcoTilling and qRT-PCR analysis. Click here for file
