# Supplementary material for: Diversity of floral regulatory genes of japonica rice cultivated at northern latitudes
Source: BMC Genomics. 2014 Feb 5;15:101. doi: 10.1186/1471-2164-15-101 (PMC3922420; doi:10.1186/1471-2164-15-101)
Supplement: Additional file 1: Table S1 — Sequences of primers used in EcoTilling and qRT-PCR analysis. [file 1471-2164-15-101-S1.docx]

**Supplemental Table S1.-** **Sequences of primers used in EcoTilling and qRT-PCR analysis**.

| **gene** | **PCR** | **primers Forward / Reverse (5’ – 3’)** |
| --- | --- | --- |
| *Ehd1* | EcoTilling | GCTTGAGGGTAGAGGAGACA / TCACAGGTTTTAGCAGGAAA |
| *Ehd1* | qRT-PCR | GAGCAAGTTGCCAGTC / CATGCACTCTGAGCCA |
| *Hd3a* | EcoTilling | TTTTACGGGTTGATAAGTTGC / ATAGGTGACCTTGAGGTTGGT |
| *DTH2* | EcoTilling | TTTTTGGCTTTTTGCACACCA / CAGCTGCTGAAAATTGCTACTTGA |
| *Hd3a* | qRT-PCR | GATGCACCAAGCCCAAGTGA / GGAACAGCACGAACACCA |
| *Hd1* | EcoTilling | CATCAGGGGGTGAGAAGAGA / ATGAACTCACGCTGTTGCTG  GCTTACACAGATTCCATCAGC / GGAGTAGTTCTAGACAATCTG |
| *RFT1* | EcoTilling | ACCCTAACCTTAGGGAGTATCTACAC / GAAGTTCCTGGTGCTGAAGTTCT |
| *RFT1* | qRT-PCR | ACCCTAACCTTAGGGAGTATCTACAC / GAAGTTCCTGGTGCTGAAGTTCT |
